# Recent advances in self‐immobilizing fluorescent probes for in vivo imaging

**DOI:** 10.1002/smo.20240031

**Published:** 2024-08-23

**Authors:** Yong Zhang, Xuemei Lv, Yafu Wang, Xueqian Chen, Jinchao Zhang, Dongdong Su

**Affiliations:** ^1^ State Key Laboratory of New Pharmaceutical Preparations and Excipients Key Laboratory of Medicinal Chemistry and Molecular Diagnosis of Ministry of Education College of Chemistry & Materials Science Chemical Biology Key Laboratory of Hebei Province Hebei University Baoding China; ^2^ School of Chemistry and Chemical Engineering Henan Normal University Xinxiang Henan China; ^3^ Department of Chemistry Center of Excellence for Environmental Safety and Biological Effects Beijing Key Laboratory for Green Catalysis and Separation Beijing University of Technology Beijing China

**Keywords:** biomarkers, fluorescent probes, in situ precise imaging, self‐immobilizing

## Abstract

In situ precise detection of bioactive molecules with high sensitivity and spatiotemporal resolution is essential for studying physiological events and disease diagnosis. The utilization of versatile fluorescent probes in fluorescence imaging offers a powerful tool for in vivo imaging of biomarkers closely associated with pathological conditions. However, the dynamic behavior leading to rapid clearance of small molecule probes from regions of interest severely compromises their potential for precise imaging. Notably, self‐immobilizing fluorescent probes that selectively recognize diseased tissues while improving in situ retention and enrichment enable accurate high‐fidelity fluorescence imaging. In this review, we aim to summarize the strategies employed for recent advances in the performance and precision of in vivo fluorescence imaging using self‐immobilizing techniques. Lastly, we discuss the prospects and potential challenges associated with self‐immobilizing fluorescent probes to promote further development and application of more delicate fluorescent probes.

## Graphical abstract

Self‐immobilizing fluorescent probes selectively recognize diseased tissues while improving in situ retention and enrichment, which overcome the rapid clearance of molecular fluorescent probes from regions of interest, enabling accurate high‐fidelity fluorescence imaging. In this review, we summarize the strategies employed for recent advancements in the performance and precision of in vivo imaging using self‐immobilizing techniques.

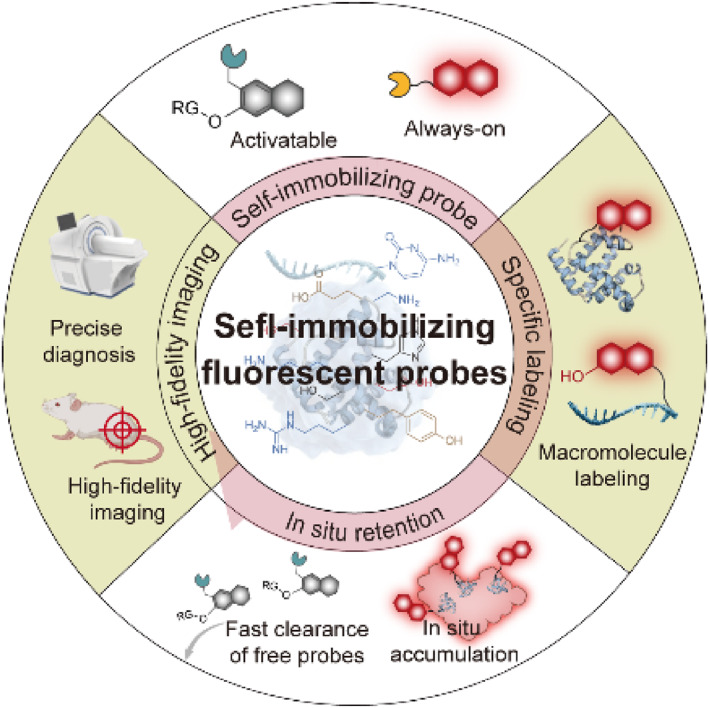

## 1 INTRODUCTION

With the rapid advancement of fluorescence imaging instruments, the utilization of versatile fluorescent probes in fluorescence imaging has become an indispensable tool in the field of biomedicine.^[1]^ Benefiting from its noninvasiveness, high sensitivity and exceptional spatiotemporal resolution, fluorescence imaging not only enables noninvasive acquisition of physiological and pathological information in vivo, but also provides an effective tool for investigating and diagnosing pathological events at an early stage, as well as evaluating drug efficacy and pharmacokinetics.^[2]^ In addition, numerous fluorescent dyes exhibit high reactive oxygen species (ROS) quantum yields under appropriate light irradiation, which manifests an indispensable tool for photodynamic therapy of tumors.^[3]^ After extensive exploration and investigation over a prolonged period, various types of fluorescent probes such as cyanine, rhodamine, BODIPY have been constructed, rendering them crucial tools for disease diagnosis and physiological research.^[4]^ Based on their sensing mode, fluorescent probes can be categorized into “always‐on” or activatable fluorescent probes; however, most are classified as “always‐on” probes due to their emission of constant bright fluorescence under suitable excitation light.^[5]^ Compared with “always‐on” fluorescent probes, activatable fluorescent probes are designed by integrating a modifiable fluorophore with a removable quenching group that allows activation of their fluorescence signal in the presence of specific biomarkers.^[6]^ These distinctive features advantageously make activatable fluorescent probes highly attractive for molecular diagnostics, especially for tumor diagnosis, since the overexpression of various active molecules and enzymes is closely associated with pathological conditions.^[7]^

However, organisms are intricate and highly dynamic physiological environments.^[8]^ Despite the capability of most current small molecule fluorescent probes to generate high‐contrast fluorescent signals in response to disease‐related biomarkers, they still encounter challenges due to their rapid clearance from regions of interest caused by the dynamic behavior of small molecules.^[9]^ These issues not only diminish the optical brightness produced by activated probes at disease regions but also result in off‐target fluorescence, severely compromising their potential for precision imaging.^[10]^ High‐dose administration is employed to enhance enrichment of fluorescent agents at disease sites, which inevitably leads to significant side effects within the body. Therefore, efficient and selective recognition of diseased tissues as well as improved in situ retention and enrichment of probes are crucial for molecular diagnostics. Notably, small molecule probes with tailorable structures allow modular design that imparts desired features, enabling improvement in metabolic behavior and sensing performance while facilitating their effective application in organisms.^[11]^ The in situ retention of imaging agents primarily relies on nanoscale‐dependent passive retention and receptor‐ligand mediated affinity interactions, which confer them with a certain degree of in situ enrichment and retention.^[12]^ However, the limited tissue penetrability of large‐sized nanomaterials and the insecure receptor‐ligand interaction do not guarantee efficient retention of imaging agents in disease tissues.^[13]^ Rao et al. proposed an in situ self‐assembly approach for small‐molecule probes that relies on intermolecular interaction forces such as π‐π interactions, hydrophobic interactions, etc., where the in situ formation of nanostructures with a slower diffusion rate contributes to aggregation and retention of imaging agents in target regions.^[14]^ Despite presenting improved retention and performance of fluorescent probes for in vivo applications, this strategy has some shortcomings including complex preparation procedures and undefined behavior during in vivo assembly, makes it necessary to conduct a long period of exploration in the further applications.

In addition to exhibiting good biocompatibility and specific recognition of disease‐related biomarkers, ideal fluorescent probes for in vivo applications, especially those administered intravenously, should also possess the following features: (1) excitation/emission wavelengths falling within the red or NIR ranges for enhanced imaging depth and bioavailability; (2) high fluorescence quantum yields that facilitate effective imaging; (3) efficient retention and accumulation within diseased tissues after systemic administration to enable high‐fidelity fluorescence imaging.

Biological macromolecule bioconjugation and functional labeling are indispensable tools for investigating physiological events and metabonomics.^[15]^ Functionalized handles, designed based on reactive residues in biological macromolecules, enable the covalent labeling of various functional molecules, such as fluorophores, biotin and activity inhibitors; for more in‐depth understanding of the ingenious mechanism of these strategies, a number of excellent reviews are recommend in the field.[^11^, ^15^, ^16^] Current chemical tools for biomacromolecule labeling mainly include activity‐based, bioorthogonal, light‐activatable and self‐labeling tag probes that specifically label biomacromolecules in biological samples.^[17]^ This facilitates the exploration of biological macromolecule functions, protein purification and medicinal development, which deepens the understanding of life science.^[18]^ Importantly, unlike highly dynamic small molecules, biological macromolecules such as proteins and nucleic acids function within unique cellular environments over extended periods. Due to their slow metabolic clearance rates, selective covalent labeling of fluorescent probes in biological macromolecules overcomes the highly dynamic behavior exhibited by small‐molecule probes, which helps to inhibit their rapid diffusion outside the regions of interest.^[19]^ Consequently, labeled probes can be retained and accumulated in disease tissues for an extended period, enabling accurate high‐fidelity fluorescence imaging in situ. Currently, numerous self‐immobilizing fluorescent probes have been developed for bioconjugation of biological macromolecules and in situ fluorescence imaging. These self‐immobilizing fluorescent probes can be broadly categorized into two types: activity‐related or specific interaction‐triggered self‐immobilizing probes that enables their efficient retention in diseased tissues. Consequently, these distinctive features facilitate overcoming the challenges posed by the highly dynamic and rapid clearance behavior exhibited by small molecule probes in vivo.

**SCHEME 1:**
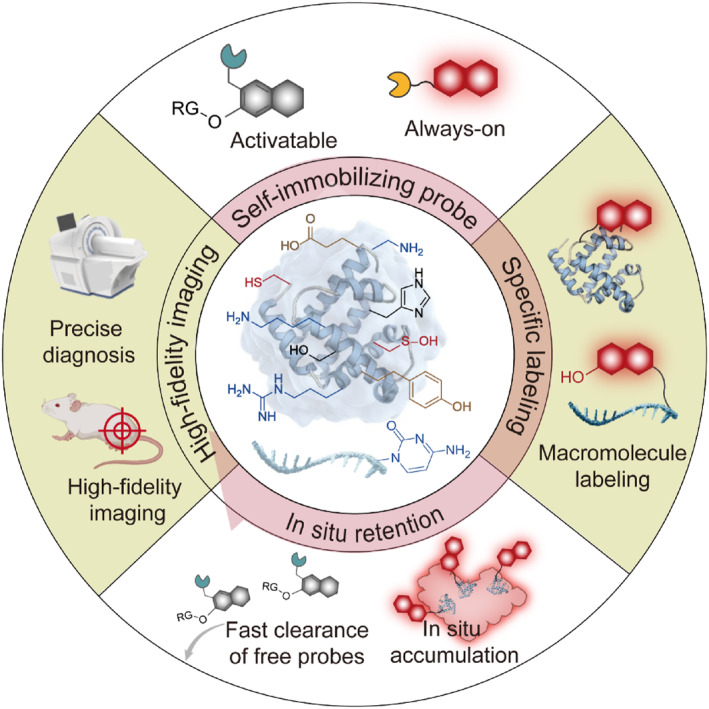
Overview of emerging self‐immobilizing fluorescent probes for precise fluorescence imaging in vivo.

Self‐immobilizing fluorescent probes exhibit long‐term retention in situ and high accumulation efficiency at lesion sites, enabling accurate diagnosis of various diseases within a complex physiological system (Scheme 1). In this review, we aim to present the current advances of selective protein labeling using fluorescent probes for in vivo imaging. A detailed overview of the latest developments in biological macromolecule labeling strategies and mechanisms for fluorescent probes is provided. Next, we rationally analyze and discuss design strategies as well as key applications of these fluorescent probes in biomarker detection and bioimaging. Finally, the advantages, prospects and future challenges associated with such probes for in vivo imaging are discussed. It is anticipated that this review will offer a comprehensive perspective on the development of retention probes in situ and promote their advancement in the fields of bioimaging.

## 2 SELF‐IMMOBILIZING FLUORESCENT PROBES FOR IN VIVO APPLICATIONS

**SCHEME 2:**
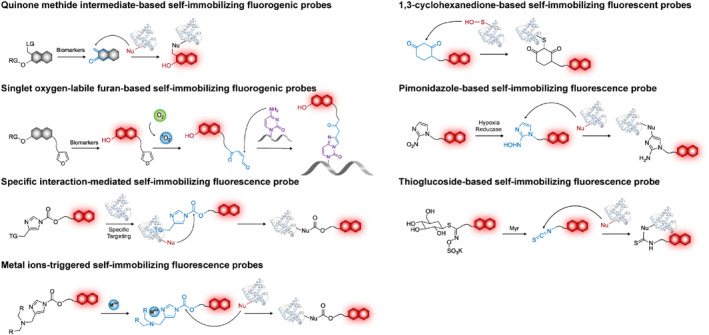
Illustration of the design mechanisms of self‐immobilizing fluorescent probes.

Self‐immobilizing fluorescent probes can be broadly categorized into two types. The first category includes activity‐related self‐immobilizing probes that generate active intermediates with short half‐lives and high electrophilicity that can be attacked by surrounding nucleophilic groups of biological macromolecules to form stable covalent bonds.^[20]^ These activity‐based self‐immobilized probes have been extensively developed for rapid labeling of biological macromolecules upon specific biomarker recognition.^[11a]^ The second category comprises specific interaction‐triggered self‐immobilizing probes, which facilitate ligand‐receptor mediated covalent tagging or selective labeling of residues (such as sulfenic acids) on biological macromolecules.^[16a]^ This process hinders high dynamics of small molecules and enables their efficient retention in diseased tissues. Recently, significant progress has been made in the development of self‐immobilizing fluorescent probes based on diverse strategies for precise imaging (Scheme 2).

### 2.1 Quinone methide intermediate‐based self‐immobilizing fluorescent probes

#### 2.1.1 (Di)fluoromethyl‐based self‐immobilizing fluorescent probes

**FIGURE 1:**
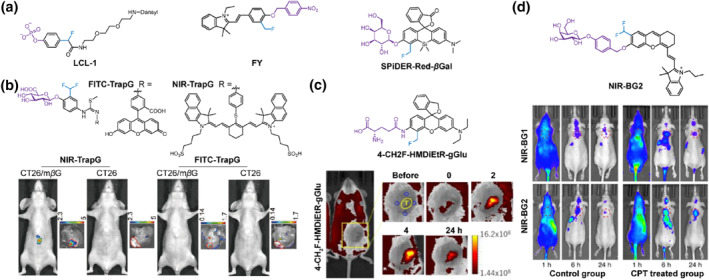
(a) Chemical structures of self‐immobilizing probes **CLC‐1**, **FY** and **SPiDER‐Red‐*β*Gal**. (b) Design of *β*‐Glu‐triggered self‐immobilizing fluorescent probes **FITC‐TrapG** and **NIR‐TrapG**, and their applications in fluorescence imaging of mice bearing *β*Glu‐overexpressed CT26/mβG or non‐*β*Glu‐expressed CT26 tumors in the liver. Reproduced with permission.^[23]^ Copyright 2012, American Chemical Society. (c) Construction of GGT activatable self‐immobilizing fluorescent probe **4‐CH**_**2**_ **F‐HMDiEtR‐gGlu** and in vivo fluorescence imaging of a PDX mouse model of pancreatic cancer after intradermal injection with **4‐CH**_**2**_ **F‐HMDiEtR‐gGlu** or control probe **gGlu‐HMRG**. Reproduced with permission.^[27]^ Copyright 2021, John Wiley and Sons. (d) Chemical structure of SA‐*β*‐Gal‐activatable self‐immobilizing fluorescent probe **NIR‐BG2**, and in vivo fluorescence imaging of CPT or non‐treated HeLa tumor‐bearing mice after intravenous injection of **NIR‐BG2** and control probe **NIR‐BG1** without self‐immobilizing moiety. Reproduced with permission.^[30]^ Copyright 2021, American Chemical Society.

In the presence of *para*‐ or *ortho*‐fluoromethyl groups with strong electron‐withdrawing features, the removal of a caged group on phenol derivatives triggers the reconstitution of aromatic rings and generates reactive quinone methides, allowing covalently labeling to surrounding nucleophiles on proteins for accurate in situ fluorescence imaging. In 2001, Lin's group reported a self‐immobilizing fluorescent probe **LCL‐1** for the covalent labeling and specific detection of protein tyrosine phosphatase‐1B (PTP‐1B) (Figure 1a).^[21]^ **LCL‐1** was composed of a phenyl phosphate moiety with a *para*‐fluoromethyl substitution and a dansyl fluorophore connected to the phenol via a short PEG2 linker. Upon selective response to PTP‐1B, **LCL‐1** generated an extremely electrophilic quinone methide intermediate that facilitated covalent labeling of PTP‐1B. Subsequently, several (di)fluoromethyl‐based self‐immobilizing fluorescent probes were developed for detecting biomolecules and proteomic analysis in living cells.[^20^, ^22^] However, the short emission wavelengths hinder further exploration in visualizing biomarkers in vivo.

In 2012, Leu's group reported two *β*‐glucuronidase (*β*Glu)‐triggered self‐immobilizing fluorescent probes, **FITC‐TrapG** and **NIR‐TrapG**, which consisted of a difluoromethylphenol‐glucuronide moiety responsible for specific labeling of *β*Glu and a high‐emission fluorescent reporter (FITC or IR‐820) (Figure 1b).^[23]^ It was observed that **FITC‐TrapG** selectively labeled *β*Glu and *β*Glu‐overexpressed CT26 cells (CT26/m*β*G) without affecting the activity of *β*Glu, while the fluorescence from non‐*β*Glu‐expressed CT26 cells was negligible. Both **FITC‐TrapG** and **NIR‐TrapG** were utilized for imaging subcutaneous CT26/m*β*G tumors in vivo. However, due to the limited penetrability of visible light, only **NIR‐TrapG** with NIR emission could be used for deep tumor imaging in the liver. Therefore, NIR fluorescent probes with enhanced penetrability and enzyme‐triggered labeling offer the capability to detect biomarker concentrations and activities in deep tissues, providing powerful tools for precise imaging and pathological studies in vivo.

Such probes achieved the labeling and detection of enzymes by utilizing the enzyme‐triggered site‐caged (di)fluoromethylphenol moiety and “always‐on” signaling reporters. However, their distribution in both diseased and normal tissues may result in inevitable background signals, thereby reducing sensitivity during in vivo imaging.^[24]^ Activatable fluorescent probes are capable of lightening indicative fluorescence upon specifically recognizing high levels of biomarkers at disease sites, providing high‐contrast fluorescent signals in specific regions.^[25]^ In 2018, Urano's group reported an *ortho*‐fluoromethyl‐substituted spiro‐silicon rhodol probe **SPiDER‐Red‐*β*Gal** for detecting *β*‐galactosidase (*β*‐Gal) in living samples (Figure 1a).^[26]^ The presence of *β*‐Gal triggered the release of the *β*‐galactoside moiety of **SPiDER‐Red‐*β*Gal** and the generation of quinone methides, enabling accurate imaging of cells with high *β*‐Gal levels at single‐cell resolution in *Drosophila melanogaster* tissue by reestablishing π‐conjugation of the xanthene ring accompanied by restoring bright red fluorescence. Following a similar strategy, a *γ*‐glutamyl‐caged fluoromethyl‐substituted fluorescent probe **4‐CH**_**2**_ **F‐HMDiEtR‐gGlu** was developed for sensitively detection of *γ*‐Glutamyltranspeptidase (GGT) and achieving long‐lasting tumor imaging (Figure 1c).^[27]^ Specific hydrolysis of GGT triggered fluorescence activation at 565 nm as well as covalent labeling of surrounding proteins, allowing durable imaging tumor with high GGT activities in vivo while maintaining a high tumor‐to‐background (T/B). This approach shows promise for tumor diagnosis and evaluation of antitumor drug efficacy. To visualize mitochondrial nitroreductase (NTR) activity, Ge's group has developed a hemicyanine‐based self‐immobilizing fluorescent probe **FY** with a fluoromethyl handle (Figure 1a).^[28]^ **FY** exhibits excellent mitochondrial localization and permanent retention after NTR‐triggered fluorescence activation and covalent labeling, achieving sensitive and accurate imaging of NTR in zebrafish.

Senescence is a physiological process occurring during the cell growth cycle and serves as an indicative marker for various diseases, including cardiovascular disease, diabetes, neurodegenerative disorders, and serves as a crucial antitumor mechanism.^[29]^ In 2021, Cui's group developed a self‐immobilizing NIR fluorescent probe **NIR‐BG2** for in vivo imaging of senescence‐associated *β*‐Gal (SA‐*β*‐Gal), which is widely used as a biomarker for cellular senescence (Figure 1d).^[30]^ **NIR‐BG2** contained a SA‐*β*‐Gal‐specific substrate‐caged hemi‐cyanine fluorophore and a difluoromethane self‐immobilizing handle. Upon intravenous administration of **NIR‐BG2** in CPT‐pretreated mice, remarkable fluorescence was observed specifically in tumor regions compared to untreated mice. Moreover, the fluorescence signal exhibited a prolonged retention time at 24 h post‐injection compared to the control probe **NIR‐BG1** without the self‐immobilizing handle. Consequently, **NIR‐BG2** exhibits significantly prolonged retention time, facilitating high‐contrast in vivo imaging after clearance of unbound probes.

#### 2.1.2 Carbamate‐based self‐immobilizing fluorescent probes

**FIGURE 2:**
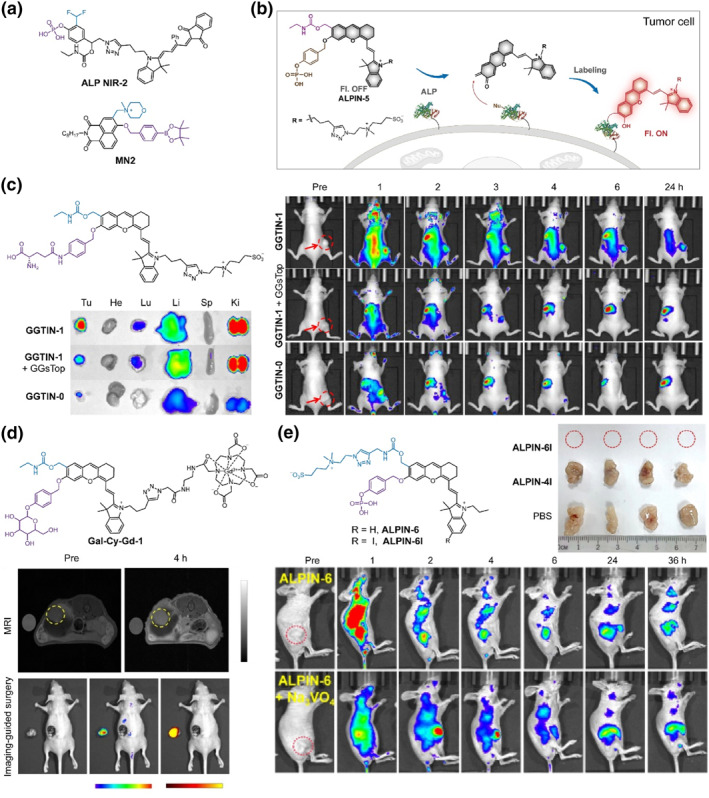
(a) Chemical structures of self‐immobilizing probes **ALP NIR‐2** and **MN2**. (b) Chemical structure and design of **ALPIN‐5**. (c) Chemical structure and design of **GGTIN‐1**, and in vivo and ex vivo fluorescence imaging of U87MG tumor‐xenografted mice with different treatment: intravenous injection with **GGTIN‐1**; intratumoral injection with GGsTop, then intravenous injection with **GGTIN‐1**; intravenous injection with control probe **GGTIN‐0** without self‐immobilizing handle. Reproduced with permission.^[33]^ Copyright 2020, American Chemical Society. (d) Chemical construction of **Gal‐Cy‐Gd‐1**, and fluorescence/MRI dual‐modal imaging‐guided surgery of orthotopic ovarian tumors after intravenous administration of **Gal‐Cy‐Gd‐1**. Reproduced with permission.^[36]^ Copyright 2023, John Wiley and Sons. (e) Structure of hydrophily tunable self‐immobilizing probe **ALPIN‐6** and **ALPIN‐6I**, and in vivo fluorescence imaging and PDT of HeLa tumor‐bearing mice with different treatment. Reproduced with permission.^[37]^ Copyright 2024, John Wiley and Sons.

The quinone methide‐based labeling strategy has been extensively investigated for self‐immobilizing fluorescence imaging; however, its insufficient labeling efficiency and non‐specificity limit its further in vivo application. In order to improve the labeling efficiency, Xie's group designed a series of self‐immobilizing fluorescent probes with multiple labeling handles, including difluoromethyl and carbamate groups (Figure 2a).^[31]^ Gel fluorescence imaging revealed that the carbamate handle offered lower nonspecific labeling compared to the difluoromethane handle. Subsequently, they employed **ALP NIR‐2**, a self‐immobilizing NIR fluorescent probe with two labeling handles, for fluorescence imaging of alkaline phosphatase (ALP) in HeLa tumor‐bearing mice. Treatment with **ALP NIR‐2** resulted in a bright fluorescence signal exclusively at the tumor site, which remained detectable for over 24 h, confirming the superiority of this self‐immobilizing probe with multiple functionalized handles.

Subsequently, Xie's group reported ALP‐activatable self‐immobilized NIR fluorescent probes featuring a carbamate as the self‐immobilized moiety (Figure 2b).^[32]^ The presence of phosphate in the phenolic hydroxyl of hemi‐cyanine resulted in negligible fluorescence within the NIR region. However, elevated levels of ALP rapidly hydrolyze the phosphate and subsequently trigger the production of highly active quinone methides intermediate, facilitating rapid labeling to surrounding proteins as well as activation of high‐contrast NIR fluorescence. Building upon similar strategy, a GGT‐activatable self‐immobilizing NIR fluorescent probe **GGTIN‐1** was proposed, which demonstrated precise visualization of GGT concentration and activity both in HepG2 cells and U87MG tumor‐xenografted mice in vivo (Figure 2c).^[33]^ Upon intratumoral injection of **GGTIN‐1**, fluorescence in tumor regions exhibited rapid enhancement with a persistent strong signal even after 48 h. In contrast, administration of the control probe **GGTIN‐0** lacking the self‐immobilizing handle led to a rapid decline in fluorescence signal within 2 h. Intravenous injection of **GGTIN‐1** also predictively generated persistent and intense NIR fluorescence specifically localized at tumor sites in U87MG tumor‐xenografted mice, exhibiting significantly higher brightness compared to the GGT inhibitor GGsTop‐treated group. In contrast, upon treatment with a control probe without self‐immobilizing handle, fluorescence signal accumulation primarily occurred within the liver due to its dynamic nature and hepatic metabolic behavior as a small molecule probe. Therefore, carbamate demonstrated exceptional potential as a self‐immobilizing handle for developing in vivo imaging probes suitable for prolonged tumor imaging.

Magnetic resonance imaging (MRI) is a widely utilized medical imaging technique that generates anatomical images with excellent tissue penetrability and spatial resolution, rendering them highly valuable for preoperative tumor detection and surgical protocol customization.^[34]^ The utilization of the quinone methide‐based strategy enables significant enhancement in the accumulation of fluorescent probes at the lesion site, facilitating precise fluorescence imaging of pathological lesions. Notably, this strategy restricts molecular rotation, leading to an enhancement in the longitudinal relaxivity of MRI contrast agents.^[35]^ Chen's group reported **Gal‐Cy‐Gd‐1** as a self‐immobilizing NIR fluorescence/MRI dual‐modal probe for detecting *β*‐Gal and guiding surgical resection of orthotopic ovarian cancer (Figure 2d).^[36]^ Following specific reaction with *β*‐Gal, the probe exhibited a remarkable 42‐fold increase in fluorescence signal, while its longitudinal relaxivity was enhanced by 1.9‐fold due to rotational restriction upon covalent binding to *β*‐Gal. Upon intravenous injection of **Gal‐Cy‐Gd‐1**, there was a substantial increase in dual‐modal signals at the tumor site compared to those observed in the liver. The combination of high‐spatial‐resolution MRI signal and highly sensitive fluorescence achieved precise detection and imaging‐guided resection of orthotopic ovarian cancer.

The high hydrophilicity of the molecules enables the diffusion of small molecule probes into tumor tissues, but also results in their rapid clearance from the regions of interest, leading to inefficient retention within tumor tissues, even with the assistance of self‐immobilizing strategy. A strategically timed alteration in the hydrophilicity of the molecule can enhance its cell permeability and retention at tumor regions. Xie's group proposed a hydrophilicity tunable strategy by further modifying the hydrophilic unit attached to the self‐immobilizing carbamate handle (Figure 2e).^[37]^ The probe **ALPIN‐6** itself exhibited good hydrophilicity, facilitating rapid diffusion into tumor regions. Subsequently, overexpressed ALP in tumor cells hydrolyzed the hydrophilic phosphate of **ALPIN‐6**, accompanied by cleavage of the hydrophilic unit, resulting in conversion of the probe into a hydrophobic feature that enhanced its in situ retention and cellular penetrability. Meanwhile, quinone methides can covalently bind to intracellular proteins, further enhancing their accumulation within tumor cells. The integration of hydrophilic switching and self‐immobilizing strategies significantly improves the accumulation of the probe at tumor regions, thereby achieving high brightness and durable imaging for HeLa tumor‐bearing mice. The introduction of halogen modifications has been proved to induce a heavy atom effect on organic dyes, which improves intersystem crossing efficiency and enhances singlet oxygen (^1^O_2_) yield (photosensitizing activity).^[38]^ Building upon the above foundation, the incorporation of an iodine atom into **ALPIN‐6** yielded a hydrophobicity tunable and self‐immobilizing photosensitizer **ALPIN‐6I**, which realized precise and efficient photodynamic therapy (PDT) in HeLa tumor‐bearing mice.

#### 2.1.3 Methyl quaternary ammonium‐based self‐immobilizing fluorescent probes

The development of a diverse range of self‐immobilizing handles has facilitated the creation of functionalized probes. In addition to (di)fluoromethyl and carbamate, Ahn's group has successfully developed a methyl quaternary ammonium‐based self‐immobilizing fluorescent probe platform, which offered a variety of probes for protein labeling triggered by light or ROS (Figure 2a).^[39]^ Then, a ROS‐activatable two‐photon fluorescent probe **MN2** was developed for imaging ROS in mouse kidney tissue. Upon treatment with **MN2**, mouse kidney tissue pretreated with hydrogen peroxide (H_2_O_2_) exhibited distinct fluorescence that was not removed using washing procedures. Therefore, the strong electron‐withdrawing methyl quaternary ammonium is suitable as a self‐immobilizing handle for efficient labeling of surrounding proteins, providing a powerful handle to develop new self‐immobilizing probes.

Therefore, quinone methide intermediate‐based self‐immobilizing fluorescent probes provide a potent tool for rapid covalent labeling on surrounding macromolecules after specifically responding to biomarkers of interest, which enables improvement of metabolic behavior of small molecules and achieves precision imaging in complex biological environments. However, it should be noted that the versatile probes usually necessitate laborious synthesis, which poses challenges for general investigations and needs to be addressed.

### 2.2 Singlet oxygen‐labile furan‐based self‐immobilizing fluorescent probes

**FIGURE 3:**
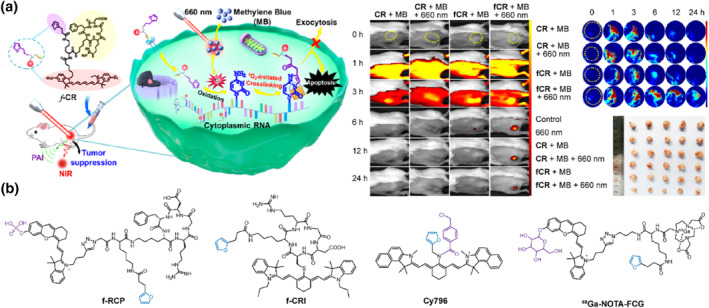
(a) Red photoinitiated RNA crosslinking probe ***f*‐CR** for prolonged fluorescence/PA dual‐modal imaging and tumors suppression. Reproduced with permission.^[42]^ Copyright 2020, American Chemical Society. (b) Chemical structures of ALP‐activated RNA crosslinking probe ***f*‐RCP**, NIR photoinitiated RNA crosslinking probe ***f*‐CRI**, H_2_S‐responsive RNA crosslinking fluorescent probe **Cy796** and *β*‐Gal‐activatable RNA crosslinking fluorescence/PET dual‐modal probe ^**68**^ **Ga‐NOTA‐FCG**.

Light serves as an external stimulus with easy‐to‐operate, high spatiotemporal resolution, making it extensively employed in photoinitiated self‐immobilizing strategy for biological macromolecule labeling.^[40]^ In 2018, Madder's group introduced a red light‐triggered furan activation strategy for RNA crosslinking that enables high spatiotemporal resolution investigation of biomolecular interactions in living cells.^[41]^ Subsequently, Shi's group presented a new attempt of this strategy in vivo fluorescence imaging and tumor therapy (Figure 3a).^[42]^ They developed a tumor‐targeting RNA crosslinking probe ***f*‐CR** by incorporating a furan handle into a cyclic‐(arginine‐glycine‐aspartic) (cRGD)‐modified cyanine fluorophore. The modified cRGD peptide facilitates specific targeting and internalization of the probe by α_*v*_β_3_ integrin‐overexpressed tumor cells.^[43]^ Upon exposure to ^1^O_2_ generated by methylene blue under 660 nm irradiation, the furan handle undergoes conversion into a reactive intermediate, enabling covalent labeling of surrounding nucleobases on RNA via a cyclic addition. Due to the promising potential of cyanine for NIR fluorescence and photoacoustic (PA) imaging,^[44]^ the probe ***f*‐CR** was employed for dual‐modal imaging of 4T1 tumor‐bearing nude mice. Upon irradiation with 660 nm light after treatment with ***f*‐CR** and methylene blue, the dual‐modal signals at the tumor site exhibited significant enhancement and remained clearly visible even after 24 h post‐irradiation. In contrast, rapid attenuation of the dual‐modal signal was observed at the tumor site when using a control probe **CR** without a furan handle, thus demonstrating excellent retention capability of the photoinitiated furan handle. Chemical modification of RNA has been demonstrated to induce aberrant expression of downstream proteins, resulting in significant apoptosis.^[45]^ Subsequently, the probe ***f*‐CR** was attempted as a novel approach for tumor suppression, unexpectedly revealing remarkable suppression of 4T1 tumors. Therefore, the photoinitiated furan‐based RNA crosslinking strategy not only enhances the precision of tumor imaging but also achieves effective tumor suppression.

However, the differences in biodistribution between the furan‐functionalized probe and methylene blue may present challenges such as low efficiency of RNA crosslinking and off‐target phototoxicity. To address this issue, Shi's group introduced a multifunctional hemi‐cyanine with NIR fluorescence emission and light‐triggered ^1^O_2_ generation (Figure 3b).^[46]^ An all‐in‐one enzyme‐activatable RNA crosslinking probe ***f*‐RCP** overcame the inconsistent biodistribution between the furan‐functionalized probe and photosensitizer. The modified cRGD peptide enabled specific targeting of ***f*‐RCP** to tumor cells, accompanied by activation of fluorescence and photoacoustic bimodal signals through pathological ALP. More importantly, under irradiation at 660 nm, ***f*‐RCP** itself can generate ^1^O_2_ that triggers RNA crosslinking and mitochondria damage, resulting in severe apoptosis of 4T1 cells and effective suppression of tumor growth. In addition, athochromic‐shift of excitation light with higher penetrability facilitated improved bioavailability of photosensitive materials.[^1^, ^47^] Based on a similar design approach, an 808 nm light triggered RNA crosslinking probe ***f*‐CRI** was developed composing a multifunctional IR780 responsible for fluorescent/PA imaging as well as in situ PDT, along with a cRGD peptide and a furan handle (Figure 3b).^[48]^ Systemic administration of ***f*‐CRI** allowed durable precise imaging of tumors along with efficient inhibition of 4T1 tumors. Therefore, the incorporation of furan‐functionalized theranostic agents addressed the challenge posed by low crosslinking efficiency while providing an effective strategy for precision diagnosis and efficient tumor suppression.

The utilization of a furan‐based RNA crosslinking strategy enables the targeted retention of small molecule agents in regions of interest, facilitating high‐contrast and durable in vivo imaging. Based on this strategy, Chen's group reported the development of a hydrogen sulfide (H_2_S)‐activated ratiometric fluorescent probe **Cy796** for the sensitive detection of H_2_S‐related colon tumors in vivo (Figure 3b).^[49]^ Under 808 nm irradiation, **Cy796** efficiently generates ^1^O_2_, leading to selective RNA crosslinking within colon cancer cells. Elevated levels of H_2_S specifically trigger leakage of the benzyl chloride ester moiety and transform **Cy796** into homologous product **Cy644**, resulting in a hypochromatic‐shift in absorption and emission spectra along with decreased ^1^O_2_ yield and increased fluorescence quantum yield. Upon administration of **Cy796**, the probe exhibited prolonged retention at the tumor site enabling accurate ratiometric detection of H_2_S in colon cancer.

Multimodal imaging probes that integrate high sensitivity, high resolution, and deep tissue imaging capabilities across different modalities facilitate a more comprehensive and precise in vivo visualization of biological events.^[50]^ Lin's group proposed a dual‐modal probe, ^**68**^ **Ga‐NOTA‐FCG**, which combines *β*‐gal activated photoinitiated RNA crosslinking fluorescence with positron emission tomography (PET) (Figure 3b).^[51]^ The chelation of radioactive ^68^Gd by NOTA serves as a PET singling reporter. This probe exhibits specific responsiveness to *β‐*Gal within lysosomes of SKOV3 cells, leading to the activation of NIR fluorescence and photoinitiated RNA crosslinking, thereby achieving high‐precision dual‐modal imaging for ovarian cancer‐bearing mice. Benefiting from the versatile design and unique features, red light‐triggered furan‐based self‐immobilizing fluorescent probes achieve efficient RNA crosslinking, representing a promising option for developing theranostic agents with high spatiotemporal resolution.

### 2.3 N‐Acyl imidazole‐based self‐immobilizing fluorescent probes

#### 2.3.1 Specific interaction‐mediated self‐immobilizing fluorescent probe

Ligand‐receptor interactions‐mediated protein labeling are commonly used methods in the investigation of physiological events and metabonomics.^[52]^ These methods commonly utilized moderately reactive electrophilic moieties, such as N‐Acyl imidazole, Dibromophenyl benzoate, N‐Sulfonyl pyridine and N‐Acyl‐N‐alkyl sulfonamide. Upon ligand‐protein specific affinity, the moderately electrophilic moieties of fluorescent probes are confined to the vicinity of the nucleophilic groups on target protein through proximity effects, thereby enhancing their reactivity and enabling labeling of fluorescent reporters on the target protein.^[53]^

**FIGURE 4:**
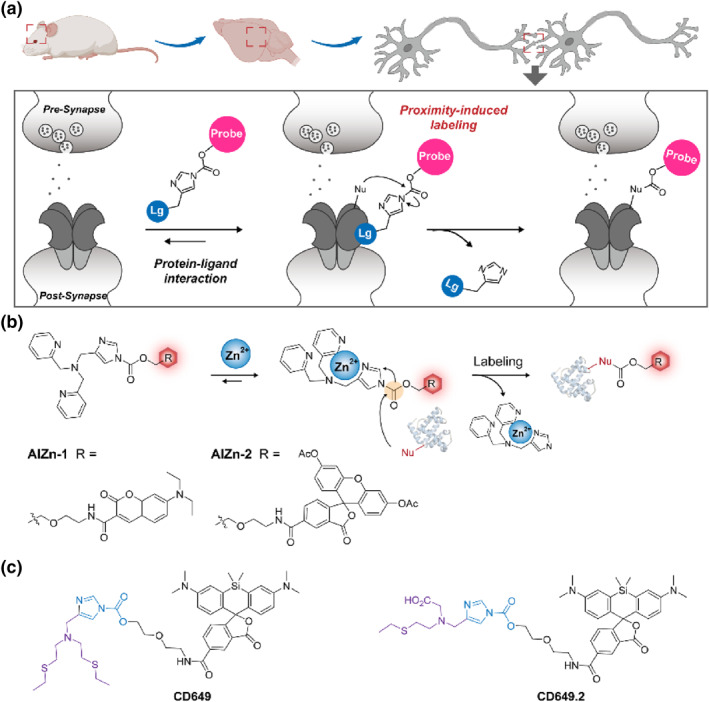
(a) Response mechanism of specific interaction‐mediated self‐immobilizing fluorescent probes for selective labeling and fluorescence imaging of various neurotransmitter receptors.^[55]^ (b) Design of Zn^2+^‐responsive self‐immobilizing fluorescent probes **AlZin‐1** and **AlZin‐2**. (c) Chemical structure of Cu‐specifically and Cu(II)‐specifically triggered self‐immobilizing fluorescent probes **CD649** and **CD649.2**.

N‐Acyl imidazole is a frequently used moderately electrophilic group that allows easy modification making it suitable for chemical labeling of proteins.^[54]^ In this section, we thereby briefly discuss the design and in vivo applications of a self‐immobilizing strategy based on specific interaction‐mediated using an example of N‐acylimidazole‐functionalized fluorescent probes. Hamachi's group has designed several ligand‐receptor‐mediated self‐immobilizing fluorescent probes consisting of an N‐acylimidazole as the electrophilic portion, cyanine with different numbers of negative charges as the reporter moiety, and specific brain receptor ligands (Figure 4a).^[55]^ Among them, the negatively charged probes selectively label various neurotransmitter receptors in the mouse brain, including N‐methyl‐D‐aspartate receptors and metabotropic glutamate receptor 1. These excellent features combined with tissue clearance procedures enabled the visualization of 3D distribution patterns of these receptors within intricate cerebral environments. Therefore, ligand‐receptor interaction‐mediated covalent conjugation offers significant advantages for highly specific labeling and visual studies of non‐enzyme proteins in intricate organisms. Notably, self‐immobilizing probes are covalently attached to the active groups of proteins in cells and in vivo for long periods of time, which can hamper the proteins functions and raise biosafety concerns, which is a double‐edged sword that must be considered in the studies of biological function and pathways.

#### 2.3.2 Metal ions‐triggered self‐immobilizing fluorescent probes

Metal ions such as copper, zinc and iron play a crucial role in maintaining normal homeostasis.^[56]^ However, elevated intracellular concentrations of these metal ions can lead to cell disorders, which further generate elevated concentrations of ROS and cellular damage.^[57]^ Despite the development of numerous small molecule fluorescent probes for real‐time visualization of metal ions, achieving high‐fidelity imaging of metal ion concentration remains challenging due to the rapid diffusion behavior exhibited by these probes.^[58]^

In 2016, Hamachi's group reported a self‐immobilizing fluorescent probe Zn^2+^, which involved linking an N‐acyl imidazole with a Zn^2+^‐specific tridentate ligand dipyridylamine (Dpa) through a methylene bond (Figure 4b).^[59]^ This chelation process reduced the electron density of the imidazole and enhanced the reactivity towards carbamate for surround protein labeling. Copper is a vital nutrient critical for signal transduction in the nervous system; however, its dyshomeostasis is associated with neuropathy and inflammation. Based on a similar chelation strategy, Chang's group designed a copper ion (Cu(I) and Cu(II))‐triggered self‐immobilizing fluorescent probe CD649 for the study of inflammation‐stimulated changes in copper concentration within living cells (Figure 4c).^[60]^ However, CD649 exhibited an indiscriminate response to both Cu(I) and Cu(II). To improve the selectivity towards Cu(II), Chang's group proposed a new Cu(II) specifically triggered self‐immobilizing fluorescent probe CD649.2 by replacing a thioether with a harder carboxylate ligand for the function investigation of Cu(II) in physiological systems (Figure 4c).^[61]^ This approach facilitates the development of novel metal ion fluorescent probes with improved sensitivity and accuracy.

### 2.4 1,3‐cyclohexanedione‐based self‐immobilizing fluorescent probes

During cellular oxidative stress, protein sulfhydryls are reversibly modified by surrounding high levels of ROS, leading to the formation of protein sulfenic acids (PSA) that play a crucial role in signaling transduction during various biological processes.^[62]^ However, PSA represents a transient oxidation species, which poses challenges for its detection using conventional means.^[63]^ In 1974, Allison's group first reported the selective reaction between dimitone and Cys‐SOH, laying the foundation for the development of probes targeting PSA detection.^[64]^

**FIGURE 5:**
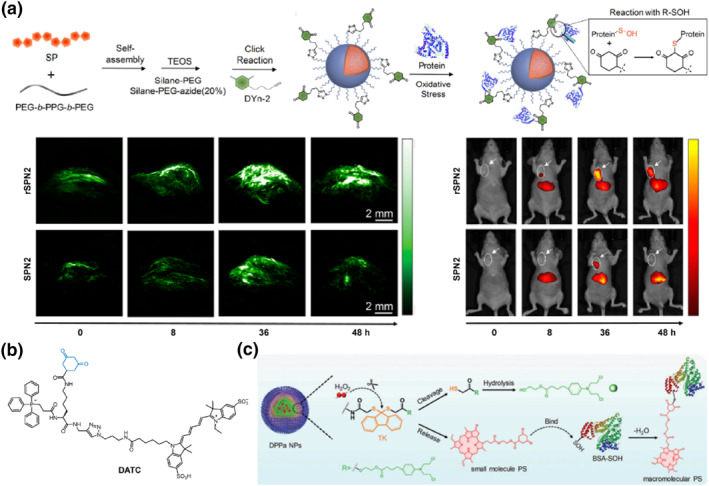
1,3‐cyclohexanedione‐based fluorescent probes. (a) Design of semiconducting polymer nanoprobes **SPN2** for fluorescence/PA dual‐modal imaging of PSA levels in HeLa xenograft tumor‐bearing mice. Reproduced with permission.^[65]^ Copyright 2017, American Chemical Society. (b) Chemical structure of mitochondrial targeting self‐immobilizing fluorescent probe **DATC**. (c) Design of H_2_O_2_‐triggered tumor‐specific phototheranostic nanomedicine **DPPa NP**. Reproduced with permission.^[68]^ Copyright 2017 John Wiley and Sons.

In 2017, Pu's group reported the development of semiconducting polymer nanoprobes **SPN2** based on 1,3‐cyclohexanedione handle for in vivo fluorescence/PA dual‐modal imaging of PSA (Figure 5a).^[65]^ The surface moiety 1,3‐cyclohexanedione of **SPN2** specifically recognized and formed covalent bonds with PSA induced by oxidative stress, enabling real‐time monitoring of changes in intracellular oxidative stress‐related PSA levels. Subsequently, the probe was applied to detect the concentration and distribution of PSA in HeLa xenograft tumor‐bearing mice. The dual‐modal signals at tumor regions exhibited an accumulative tendency after probe injection and reached its plateau at 36 h post‐injection. During tumor imaging, integration of fluorescence imaging with high signal‐to‐noise ratio (SNR) and PA imaging with high resolution enabled accurate tumor detection over an extended imaging window.

Studies have demonstrated that the levels of ROS in tumors are significantly higher than those in normal tissues, with approximately 90% of intracellular ROS being generated by mitochondria.^[66]^ As a result, the mitochondrial PSA serves as a reliable reference for early tumor diagnosis. In light of this, Shi's group reported a mitochondrial PSA‐immobilized NIR fluorescent probe **DATC**, which was composed of a NIR fluorescent reporter Cy5, a mitochondrial‐targeted cationic triphenylphosphate and a PSA‐specific 1,3‐cyclohexanedione (Figure 5b).^[67]^ **DATC** showed excellent localization within mitochondria and displayed high affinity towards PSA‐enriched tumor cells, which led to significant prolongation of **DATC** retention at tumor regions and achieved prolonged visualization in 4T1‐bearing mice.

Building upon the elevated levels of PSA in tumors, Fan's group developed tumor‐specific phototherapeutic nanoparticles **DPPa NP** for retention‐enhanced photodynamic/chemo combination therapy (Figure 5c).^[68]^ The nanoscale nature facilitated the accumulation of **DPPa NPs** in tumor tissues. Upon stimulation with pathological levels of H_2_O_2_, these nanoparticles released antitumor chlorambucil and 1,3‐cyclohexanedione‐modified Ce6, allowing the retention and accumulation of Ce6 within the tumor after reaction with PSA. The ROS generated by Ce6 accelerated the cargo release from **DPPa NPs** and further enhanced the efficacy of tumor‐specific photodynamic/therapy combined chemotherapy. Benefiting from protein‐labeling retention mechanisms, self‐amplification effects and photodynamic/chemo combination therapy, **DPPa NPs** presented an excellent therapeutic effect in a 4T1 xenografted mouse model, achieving impressive tumor inhibition rates up to 98.5%. Therefore, the specific interaction of PSA‐1,3‐cyclohexanedione offers an efficient strategy for developing functionalized fluorescent probes with prolonged tumor retention and an expanded PDT treatment window, thereby providing a viable approach to effective tumor imaging and treatment.

### 2.5 Other reactive intermediates‐based self‐immobilizing fluorescent probes

#### 2.5.1 Thioglucoside‐based self‐immobilizing fluorescent probe

**FIGURE 6:**
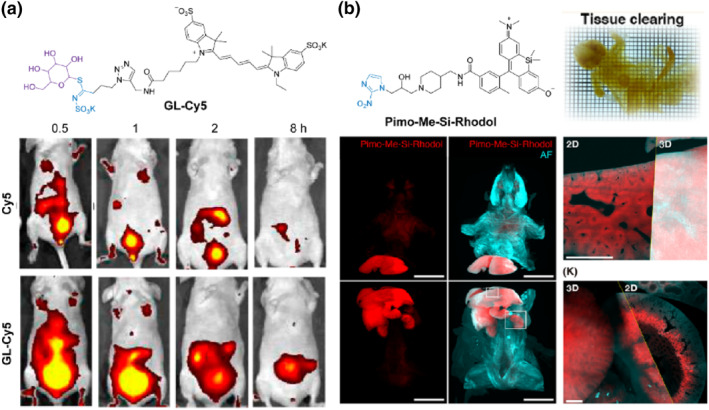
(a) Chemical structure of thioglucosides‐based self‐immobilizing fluorescent probe **GLS‐Cy5** and in vivo imaging of Myr‐expressing *B. thetaiotaomicron* in mice. Reproduced with permission.^[70]^ Copyright 2024, American Chemical Society. (b) Chemical structure of hypoxia reductase‐triggered self‐immobilizing fluorescent probe **Pimo‐Me‐SiRhodol** and its applications for the whole‐body imaging of hypoxic conditions with single cell resolution. Reproduced with permission.^[73]^ Copyright 2024, American Chemical Society.

Black Mustardase (Myr) is a *β*‐thioglucosidase that enables the conversion of thioglucosides into isothiocyanates, which serve as reactive electrophilic reagents for biolabeling purposes.^[69]^ In 2024, Xing's group reported a glucosinolate‐based self‐immobilizing fluorescent probe **GLS‐Cy5** for MYR‐triggered in situ labeling and MYR‐associated intestinal bacterial imaging (Figure 6a).^[70]^ After injection of **GLS‐Cy5**, a distinct fluorescent signal was observed in the abdomen of MYR‐expressing *B. thetaiotaomicron*‐treated mice, significantly stronger than that of control probe **Cy5**‐treated mice. The enhanced and prolonged fluorescent signals exhibited by **GLS‐Cy5** within biological environments demonstrated its great potential for the real‐time imaging of intestinal bacteria.

#### 2.5.2 Pimonidazole‐based self‐immobilizing fluorescent probe

The limited penetrability of fluorescent probes restricts their application to high‐resolution visualization of the concentration and distribution of biomarkers within the entire body or organs.^[71]^ Tissue clearing techniques have demonstrated the single‐cell‐resolved imaging of deep tissues by reducing the refractive index difference between tissue components, thereby facilitating further investigation into biological events.^[72]^ However, small molecule fluorescent probes are prone to removal during tissue clearing. Therefore, fluorescent probes that can covalently label biological macromolecules are well‐suited to tissue clearance‐mediated in vivo imaging.

Sando's group reported a self‐immobilizing fluorescent platform triggered by hypoxia reductase with pimonidazole as a functionalized handle.^[73]^ Pimonidazole‐based probes can be selectively reduced by intracellular reductases under hypoxic conditions to reactive intermediates, allowing for covalent labeling of nucleophilic residues on macromolecules (Figure 6b). After conducting compatibility studies between dyes and refractive index matching solutions, three fluorescent probes Pimo‐Si‐Rhodamine, Pimo‐Me‐SiRhodol and Pimo‐BODIPY were proposed for visualizing hypoxic conditions throughout the whole body by integration with tissue clearance. Following injection with Pimo‐Me‐Si‐Rhodol, tissue clearance enabled transparent and accurate observation of hypoxic conditions in deep tissues of mice at single cell resolution, providing a referenceable method for studying different biomarkers in chemical biology.

## 3 SUMMARY AND FUTURE PERSPECTIVES

Self‐immobilizing strategies have made significant advancements in improving the retention ability of small molecule probes within the focal regions, leading to more accurate in vivo fluorescence imaging. Various self‐immobilizing strategies with excellent selectivity and high labeling efficiency provide robust technical support for extending the observation window and improving the accuracy of probe localization. In recent years, the progress of self‐immobilizing fluorescent probes has greatly enhanced the effectiveness in disease diagnosis and therapy through in vivo applications. These advances not only deepen our understanding of designing fluorescent probes but also indicate the development direction to face future clinical opportunities and challenges.

(1) The complex biological microenvironment contains numerous molecules such as ions, small molecules, biological macromolecules, as well as highly reactive species.^[8]^ Although the mechanisms of self‐immobilizing strategies have been clearly elucidated in vitro, understanding their dynamic mechanism and behavior in vivo remains challenging. In addition, self‐immobilizing probes have been demonstrated to generate intermediates with short half‐lives and high activity that may bind to free small molecules, resulting in limited selectivity and retention efficiency. Therefore, there is an urgent need for advanced techniques that can analyze and define in vivo labeling mechanisms of self‐immobilization to provide a new guidance for improving the selectivity and efficiency of labeling biological macromolecules in situ.
(2) At present, most in vivo self‐immobilizing fluorescent probes with red and NIR‐I emission exhibit high sensitivity and spatial resolution, enabling high‐fidelity imaging in vivo. However, their limited tissue penetration depth (<1 cm) poses a challenge for obtaining comprehensive information on deep tissues and lesions for accurate diagnosis, impeding their transition to clinical application.[^24^, ^47^] The development of the next generation of in vivo self‐immobilizing fluorescent probes by incorporating NIR II fluorophores is expected to expand their in vivo application in studying disease progression and acquiring more valuable biological information.
(3) Personalized precision medicine is the pursuit direction in modern medicine as it not only enhances treatment efficacy but also minimizes side effects and medical resource wastage.^[74]^ At present, nanotheranostic agents integrate multiple functional components of drugs and imaging agents, showing extreme research value in the treatment of diseases, especially tumors. Taking advantage of these unique features, the versatile nanoplatforms enable synergistic therapy, evaluation of drug delivery efficiency and real‐time monitoring of therapeutic effect. On this basis, nanotheranostic platforms that utilize self‐immobilizing strategies offer great prospects for achieving precision medicine through real‐time monitoring of therapeutic effects using an in vivo fluorescence imaging system. Therefore, the development of theranostic probes based on self‐immobilizing strategies holds significant potential for improving the treatment efficacy and cure rates of tumor medicine.

In summary, although considerable progress has been made regarding the accurate in vivo applications of self‐immobilizing fluorescent probes, they are still at a basic research stage with numerous undefined challenges that need to be explored and overcome before they can be applied to modern clinical medicine. With continuous developments and deeper exploration into in vivo imaging technology, we anticipate that self‐immobilizing fluorescent probes will become a promising tool for future disease theranostics.

## CONFLICT OF INTEREST STATEMENT

The authors declare no conflicts of interest.

## Data Availability

Data sharing not applicable.

## References

[1] a) M. Gao , F. Yu , C. Lv , J. Choo , L. Chen , Chem. Soc. Rev. 2017, 46, 2237; 28319221 10.1039/c6cs00908e

[2] a) Y. Geng , Z. Wang , J. Zhou , M. Zhu , J. Liu , T. D. James , Chem. Soc. Rev. 2023, 52, 3873; 37190785 10.1039/d2cs00172a

[3] a) T. C. Pham , V.‐N. Nguyen , Y. Choi , S. Lee , J. Yoon , Chem. Rev. 2021, 121, 13454; 34582186 10.1021/acs.chemrev.1c00381

[4] a) R. Weinstain , T. Slanina , D. Kand , P. Klán , Chem. Rev. 2020, 120, 13135; 33125209 10.1021/acs.chemrev.0c00663 PMC7833475

[5] a) D. Su , X. Chen , Y. Zhang , X. Gao , TrAC, Trends Anal. Chem. 2020, 133, 116112;

[6] a) Y. Zhang , G. Zhang , Z. Zeng , K. Pu , Chem. Soc. Rev. 2022, 51, 566; 34928283 10.1039/d1cs00525a

[7] a) X. Wang , Q. Ding , R. R. Groleau , L. Wu , Y. Mao , F. Che , O. Kotova , E. M. Scanlan , S. E. Lewis , P. Li , B. Tang , T. D. James , T. Gunnlaugsson , Chem. Rev. 2024, 124, 7106; 38760012 10.1021/acs.chemrev.3c00776 PMC11177268

[8] Y. Fan , Y. Wu , J. Hou , P. Wang , X. Peng , G. Ge , Coord. Chem. Rev. 2023, 480, 215020.

[9] a) J. Huang , K. Pu , Angew. Chem., Int. Ed. 2020, 59, 11717; 10.1002/anie.202001783 32134156

[10] a) K. Li , Y. Lyu , Y. Huang , S. Xu , H.‐W. Liu , L. Chen , T.‐B. Ren , M. Xiong , S. Huan , L. Yuan , X.‐B. Zhang , W. Tan , Proc. Natl. Acad. Sci. U. S. A. 2021, 118, e2018033118; 33602816 10.1073/pnas.2018033118 PMC7923636

[11] a) K. Li , S. Xu , M. Xiong , S.‐Y. Huan , L. Yuan , X.‐B. Zhang , Chem. Soc. Rev. 2021, 50, 11766; 34570124 10.1039/d1cs00408e

[12] a) A. L. Antaris , H. Chen , K. Cheng , Y. Sun , G. Hong , C. Qu , S. Diao , Z. Deng , X. Hu , B. Zhang , X. Zhang , O. K. Yaghi , Z. R. Alamparambil , X. Hong , Z. Cheng , H. Dai , Nat. Mater. 2016, 15, 235; 26595119 10.1038/nmat4476

[13] Y. Wang , J. Weng , X. Wen , Y. Hu , D. Ye , Biomater. Sci. 2021, 9, 406. 32627767 10.1039/d0bm00895h

[14] a) R. Yan , Y. Hu , F. Liu , S. Wei , D. Fang , A. J. Shuhendler , H. Liu , H. Y. Chen , D. Ye , J. Am. Chem. Soc. 2019, 141, 10331; 31244188 10.1021/jacs.9b03649

[15] a) P. Chauhan , R. V , M. Kumar , R. Molla , S. D. Mishra , S. Basa , V. Rai , Chem. Soc. Rev. 2024, 53, 380; 38095227 10.1039/d3cs00715d

[16] a) M. Minoshima , S. I. Reja , R. Hashimoto , K. Iijima , K. Kikuchi , Chem. Rev. 2024, 124, 6198; 38717865 10.1021/acs.chemrev.3c00549

[17] a) H. Fang , B. Peng , S. Y. Ong , Q. Wu , L. Li , S. Q. Yao , Chem. Sci. 2021, 12, 8288; 34221311 10.1039/d1sc01359a PMC8221178

[18] a) X. Zhang , Q. Tang , J. Sun , Y. Guo , S. Zhang , S. Liang , P. Dai , X. Chen , Sci. Adv. 2023, 9, eadg6388; 37235653 10.1126/sciadv.adg6388 PMC10219591

[19] a) S. Uchinomiya , T. Nagaura , M. Weber , Y. Matsuo , N. Zenmyo , Y. Yoshida , A. Tsuruta , S. Koyanagi , S. Ohdo , N. Matsunaga , A. Ojida , J. Am. Chem. Soc. 2023, 145, 8248; 37011039 10.1021/jacs.3c02043

[20] D. H. Kwan , H.‐M. Chen , K. Ratananikom , S. M. Hancock , Y. Watanabe , P. T. Kongsaeree , A. L. Samuels , S. G. Withers , Angew. Chem., Int. Ed. 2011, 50, 300. 10.1002/anie.201005705 21184404

[21] L.‐C. Lo , T.‐L. Pang , C.‐H. Kuo , Y.‐L. Chiang , H.‐Y. Wang , J.‐J. Lin , J. Proteome Res. 2002, 1, 35. 12643524 10.1021/pr015506a

[22] a) T. Doura , M. Kamiya , F. Obata , Y. Yamaguchi , T. Y. Hiyama , T. Matsuda , A. Fukamizu , M. Noda , M. Miura , Y. Urano , Angew. Chem., Int. Ed. 2016, 55, 9620; 10.1002/anie.201603328 27400827

[23] T.‐C. Cheng , S. R. Roffler , S.‐C. Tzou , K.‐H. Chuang , Y.‐C. Su , C.‐H. Chuang , C.‐H. Kao , C.‐S. Chen , I. H. Harn , K.‐Y. Liu , T.‐L. Cheng , Y.‐L. Leu , J. Am. Chem. Soc. 2012, 134, 3103. 22239495 10.1021/ja209335z

[24] C. Chen , R. Tian , Y. Zeng , C. Chu , G. Liu , Bioconjugate Chem. 2020, 31, 276. 10.1021/acs.bioconjchem.9b00734 31935072

[25] Y. Tan , L. Zhang , K. H. Man , R. Peltier , G. Chen , H. Zhang , L. Zhou , F. Wang , D. Ho , S. Q. Yao , Y. Hu , H. Sun , ACS Appl. Mater. Interfaces 2017, 9, 6796. 28139117 10.1021/acsami.6b14176

[26] H. Ito , Y. Kawamata , M. Kamiya , K. Tsuda‐Sakurai , S. Tanaka , T. Ueno , T. Komatsu , K. Hanaoka , S. Okabe , M. Miura , Y. Urano , Angew. Chem., Int. Ed. 2018, 57, 15702. 10.1002/anie.201808670 30255610

[27] R. Obara , M. Kamiya , Y. Tanaka , A. Abe , R. Kojima , T. Kawaguchi , M. Sugawara , A. Takahashi , T. Noda , Y. Urano , Angew. Chem., Int. Ed. 2021, 60, 2125. 10.1002/anie.202013265 33096584

[28] S. Wang , W. Tan , W. Lang , H. Qian , S. Guo , L. Zhu , J. Ge , Anal. Chem. 2022, 94, 7272. 35549110 10.1021/acs.analchem.2c00512

[29] B. Lozano‐Torres , A. Estepa‐Fernández , M. Rovira , M. Orzáez , M. Serrano , R. Martínez‐Máñez , F. Sancenón , Nat. Revi. Chem. 2019, 3, 426.

[30] J. Liu , X. Ma , C. Cui , Z. Chen , Y. Wang , P. R. Deenik , L. Cui , J. Med. Chem. 2021, 64, 17969. 34752102 10.1021/acs.jmedchem.1c01313 PMC10880455

[31] H. Song , Y. Li , Y. Chen , C. Xue , H. Xie , Chem. Eur J. 2019, 25, 13994. 31506999 10.1002/chem.201903458

[32] Y. Li , H. Song , C. Xue , Z. Fang , L. Xiong , H. Xie , Chem. Sci. 2020, 11, 5889. 32874510 10.1039/d0sc01273d PMC7449546

[33] Y. Li , C. Xue , Z. Fang , W. Xu , H. Xie , Anal. Chem. 2020, 92, 15017. 33141566 10.1021/acs.analchem.0c02954

[34] J. Wahsner , E. M. Gale , A. Rodríguez‐Rodríguez , P. Caravan , Chem. Rev. 2019, 119, 957. 30350585 10.1021/acs.chemrev.8b00363 PMC6516866

[35] H.‐D. Xu , X. Cheng , X. Sun , P. Chen , W. Zhan , X. Liu , X. Wang , B. Hu , G. Liang , Nano Lett. 2023, 23, 6178. 37363812 10.1021/acs.nanolett.3c01787

[36] Q. Yu , L. Zhang , M. Jiang , L. Xiao , Y. Xiang , R. Wang , Z. Liu , R. Zhou , M. Yang , C. Li , M. Liu , X. Zhou , S. Chen , Angew. Chem., Int. Ed. 2023, 62, e202313137. 10.1002/anie.202313137 37766426

[37] Y. Li , C. Zhang , Q. Wu , Y. Peng , Y. Ding , Z. Zhang , X. Xu , H. Xie , Angew. Chem., Int. Ed. 2024, 63, e202317773. 10.1002/anie.202317773 38116827

[38] J. Zhao , K. Xu , W. Yang , Z. Wang , F. Zhong , Chem. Soc. Rev. 2015, 44, 8904. 26465741 10.1039/c5cs00364d

[39] Y. L. Jung , S. Sarkar , J. Ha , S. B. Park , K. H. Ahn , Bioconjugate Chem. 2022, 33, 1543. 10.1021/acs.bioconjchem.2c00297 35900309

[40] a) W. P. Heal , T. H. T. Dang , E. W. Tate , Chem. Soc. Rev. 2011, 40, 246; 20886146 10.1039/c0cs00004c

[41] N. De Laet , E. M. Llamas , A. Madder , Chem. Eur J. 2018, 2, 575.

[42] S. Ye , C. Cui , X. Cheng , M. Zhao , Q. Mao , Y. Zhang , A. Wang , J. Fang , Y. Zhao , H. Shi , J. Am. Chem. Soc. 2020, 142, 21502. 33306393 10.1021/jacs.0c10755

[43] S. Kunjachan , R. Pola , F. Gremse , B. Theek , J. Ehling , D. Moeckel , B. Hermanns‐Sachweh , M. Pechar , K. Ulbrich , W. E. Hennink , G. Storm , W. Lederle , F. Kiessling , T. Lammers , Nano Lett. 2014, 14, 972. 24422585 10.1021/nl404391r PMC3940962

[44] a) C. Li , C. Liu , Y. Fan , X. Ma , Y. Zhan , X. Lu , Y. Sun , RSC Chem. Biol. 2021, 2, 743; 34458809 10.1039/d0cb00225a PMC8341990

[45] a) K. D. Warner , C. E. Hajdin , K. M. Weeks , Nat. Rev. Drug Discov. 2018, 17, 547; 29977051 10.1038/nrd.2018.93 PMC6420209

[46] J. Fang , Y. Feng , Y. Zhang , A. Wang , J. Li , C. Cui , Y. Guo , J. Zhu , Z. Lv , Z. Zhao , C. Xu , H. Shi , J. Am. Chem. Soc. 2022, 144, 23061. 36503221 10.1021/jacs.2c10409

[47] Z. Lei , C. Sun , P. Pei , S. Wang , D. Li , X. Zhang , F. Zhang , Angew. Chem., Int. Ed. 2019, 58, 8166. 10.1002/anie.201904182 31008552

[48] Y. Feng , J. Fang , Y. Zhao , S. Ye , A. Wang , Y. Zhang , J. Zhu , J. Li , Z. Lv , Z. Zhao , H. Shi , Angew. Chem., Int. Ed. 2023, 62, e202218969. 10.1002/anie.202218969 36912594

[49] Z. Lu , J. Tan , Y. Wu , J. You , X. Xie , Z. Zhang , Z. Li , L. Chen , Anal. Chem. 2023, 95, 17089. 37940603 10.1021/acs.analchem.3c04033

[50] a) R. Chen , W. Li , R. Li , S. Ai , H. Zhu , W. Lin , Chin. Chem. Lett. 2023, 34, 107845;

[51] J. Fang , Q. Liu , Y. Liu , K. Li , L. Qiu , H. Xi , S. Cai , P. Zou , J. Lin , Anal. Chem. 2024, 96, 1707. 38241523 10.1021/acs.analchem.3c04845

[52] K. Shiraiwa , R. Cheng , H. Nonaka , T. Tamura , I. Hamachi , Cell Chem. Biol. 2020, 27, 970. 32679042 10.1016/j.chembiol.2020.06.016

[53] S. Kiyonaka , S. Sakamoto , S. Wakayama , Y. Morikawa , M. Tsujikawa , I. Hamachi , ACS Chem. Biol. 2018, 13, 1880. 29437380 10.1021/acschembio.7b01042

[54] T. Mino , S. Sakamoto , I. Hamachi , Biosci., Biotechnol., Biochem. 2021, 85, 53. 33577657 10.1093/bbb/zbaa026

[55] H. Nonaka , S. Sakamoto , K. Shiraiwa , M. Ishikawa , T. Tamura , K. Okuno , T. Kondo , S. Kiyonaka , E. A. Susaki , C. Shimizu , H. R. Ueda , W. Kakegawa , I. Arai , M. Yuzaki , I. Hamachi , Proc. Natl. Acad. Sci. U. S. A. 2024, 121, e2313887121. 38294939 10.1073/pnas.2313887121 PMC10861872

[56] a) A. V. Davis , T. V. O'Halloran , Nat. Chem. Biol. 2008, 4, 148; 18277969 10.1038/nchembio0308-148 PMC2265432

[57] a) L. M. Gaetke , C. K. Chow , Toxicology 2003, 189, 147; 12821289 10.1016/s0300-483x(03)00159-8

[58] a) M. T. Morgan , P. Bagchi , C. J. Fahrni , J. Am. Chem. Soc. 2011, 133, 15906; 21916472 10.1021/ja207004v PMC3388948

[59] T. Miki , M. Awa , Y. Nishikawa , S. Kiyonaka , M. Wakabayashi , Y. Ishihama , I. Hamachi , Nat. Methods 2016, 13, 931. 27617391 10.1038/nmeth.3998

[60] S. Lee , C. Y.‐S. Chung , P. Liu , L. Craciun , Y. Nishikawa , K. J. Bruemmer , I. Hamachi , K. Saijo , E. W. Miller , C. J. Chang , J. Am. Chem. Soc. 2020, 142, 14993. 32815370 10.1021/jacs.0c05727 PMC7877313

[61] A. T. Pezacki , C. D. Matier , X. Gu , E. Kummelstedt , S. E. Bond , L. Torrente , K. L. Jordan‐Sciutto , G. M. DeNicola , T. A. Su , D. C. Brady , C. J. Chang , Proc. Natl. Acad. Sci. U. S. A. 2022, 119, e2202736119. 36252013 10.1073/pnas.2202736119 PMC9621372

[62] a) Z. Li , T. E. Forshaw , R. J. Holmila , S. A. Vance , H. Wu , L. B. Poole , C. M. Furdui , S. B. King , Chem. Res. Toxicol. 2019, 32, 526; 30784263 10.1021/acs.chemrestox.8b00385 PMC6719313

[63] L. J. Alcock , B. L. Oliveira , M. J. Deery , T. L. Pukala , M. V. Perkins , G. J. L. Bernardes , J. M. Chalker , ACS Chem. Biol. 2019, 14, 594. 30893551 10.1021/acschembio.8b01104

[64] L. V. Benitez , W. S. Allison , J. Biol. Chem. 1974, 249, 6234. 4371119

[65] Y. Lyu , X. Zhen , Y. Miao , K. Pu , ACS Nano 2017, 11, 358. 27997794 10.1021/acsnano.6b05949

[66] a) Y. Cheng , J. Dai , C. Sun , R. Liu , T. Zhai , X. Lou , F. Xia , Angew. Chem., Int. Ed. 2018, 57, 3123; 10.1002/anie.201712803 29383811

[67] Y. Gao , R. Sun , M. Zhao , J. Ding , A. Wang , S. Ye , Y. Zhang , Q. Mao , W. Xie , G. Ma , H. Shi , Anal. Chem. 2020, 92, 6977. 32314575 10.1021/acs.analchem.9b05855

[68] S. Diao , Y. Liu , Z. Guo , Z. Xu , J. Shen , W. Zhou , C. Xie , Q. Fan , Adv. Healthcare Mater. 2023, 12, 2301732. 10.1002/adhm.202301732 37548967

[69] X. Wu , H. Huang , H. Childs , Y. Wu , L. Yu , P. R. Pehrsson , Annu. Rev. Food Sci. Technol. 2021, 12, 485. 33467908 10.1146/annurev-food-070620-025744

[70] W. Lang , D. Shu , S. Liu , C. Sun , H. Liu , Q. Huang , G. Mao , S. Yang , B. Xing , J. Org. Chem. 2024. 10.1021/acs.joc.3c02848 38607989

[71] C. T. Inglut , B. Gaitan , D. Najafali , I. A. Lopez , N. P. Connolly , S. Orsila , R. Perttilä , G. F. Woodworth , Y. Chen , H.‐C. Huang , Photochem. Photobiol. 2020, 96, 301. 31441057 10.1111/php.13155 PMC7035972

[72] a) E. A. Susaki , C. Shimizu , A. Kuno , K. Tainaka , X. Li , K. Nishi , K. Morishima , H. Ono , K. L. Ode , Y. Saeki , K. Miyamichi , K. Isa , C. Yokoyama , H. Kitaura , M. Ikemura , T. Ushiku , Y. Shimizu , T. Saito , T. C. Saido , M. Fukayama , H. Onoe , K. Touhara , T. Isa , A. Kakita , M. Shibayama , H. R. Ueda , Nat. Commun. 2020, 11, 1982; 32341345 10.1038/s41467-020-15906-5 PMC7184626

[73] D. M. Sakamoto , I. Tamura , B. Yi , S. Hasegawa , Y. Saito , N. Yamada , Y. Takakusagi , S. I. Kubota , M. Kobayashi , H. Harada , K. Hanaoka , M. Taki , M. Nangaku , K. Tainaka , S. Sando , ACS Nano 2024, 18, 5167. 38301048 10.1021/acsnano.3c12716

[74] a) Y. Wang , Y. Hu , D. Ye , Angew. Chem., Int. Ed. 2022, 61, e202209512; 10.1002/anie.202209512 36151870

